# Cerebrospinal Fluid Biomarker and Brain Biopsy Findings in Idiopathic Normal Pressure Hydrocephalus

**DOI:** 10.1371/journal.pone.0091974

**Published:** 2014-03-17

**Authors:** Okko T. Pyykkö, Miikka Lumela, Jaana Rummukainen, Ossi Nerg, Toni T. Seppälä, Sanna-Kaisa Herukka, Anne M. Koivisto, Irina Alafuzoff, Lakshman Puli, Sakari Savolainen, Hilkka Soininen, Juha E. Jääskeläinen, Mikko Hiltunen, Henrik Zetterberg, Ville Leinonen

**Affiliations:** 1 Neurosurgery of NeuroCenter, Kuopio University Hospital, Kuopio, Finland; 2 Neurology of NeuroCenter, Kuopio University Hospital, Kuopio, Finland; 3 Department of Neurology, Institute of Clinical Medicine, University of Eastern Finland, Kuopio, Finland; 4 Department of Pathology, Kuopio University Hospital, Kuopio, Finland; 5 Department of Immunology, Genetics and Pathology, Uppsala University, Uppsala, Sweden; 6 Clinical Neurochemistry Laboratory, Department of Psychiatry and Neurochemistry, The Sahlgrenska Academy, University of Gothenburg, Gothenburg, Sweden; Georgetown University Medical Center, United States of America

## Abstract

**Background:**

The significance of amyloid precursor protein (APP) and neuroinflammation in idiopathic normal pressure hydrocephalus (iNPH) and Alzheimer's disease (AD) is unknown.

**Objective:**

To investigate the role of soluble APP (sAPP) and amyloid beta (Aβ) isoforms, proinflammatory cytokines, and biomarkers of neuronal damage in the cerebrospinal fluid (CSF) in relation to brain biopsy Aβ and hyperphosphorylated tau (HPτ) findings.

**Methods:**

The study population comprised 102 patients with possible NPH with cortical brain biopsies, ventricular and lumbar CSF samples, and DNA available. The final clinical diagnoses were: 53 iNPH (91% shunt-responders), 26 AD (10 mixed iNPH+AD), and 23 others. Biopsy samples were immunostained against Aβ and HPτ. CSF levels of AD-related biomarkers (Aβ42, p-tau, total tau), non-AD-related Aβ isoforms (Aβ38, Aβ40), sAPP isoforms (sAPPα, sAPPβ), proinflammatory cytokines (several interleukins (IL), interferon-gamma, monocyte chemoattractant protein-1, tumor necrosis factor-alpha) and biomarkers of neuronal damage (neurofilament light and myelin basic protein) were measured. All patients were genotyped for *APOE*.

**Results:**

Lumbar CSF levels of sAPPα were lower (*p*<0.05) in patients with shunt-responsive iNPH compared to non-iNPH patients. sAPPβ showed a similar trend (*p* = 0.06). CSF sAPP isoform levels showed no association to Aβ or HPτ in the brain biopsy. Quantified Aβ load in the brain biopsy showed a negative correlation with CSF levels of Aβ42 in ventricular (*r* = −0.295, *p* = 0.003) and lumbar (*r* = −0.356, *p* = 0.01) samples, while the levels of Aβ38 and Aβ40 showed no correlation. CSF levels of proinflammatory cytokines and biomarkers of neuronal damage did not associate to the brain biopsy findings, diagnosis, or shunt response. Higher lumbar/ventricular CSF IL-8 ratios (*p*<0.001) were seen in lumbar samples collected after ventriculostomy compared to the samples collected before the procedure.

**Conclusions:**

The role of sAPP isoforms in iNPH seems to be independent from the amyloid cascade. No neuroinflammatory background was observed in iNPH or AD.

## Introduction

Idiopathic normal pressure hydrocephalus (iNPH) is a progressive neurodegenerative disorder of unknown etiology in the elderly presenting with gait disorder, cognitive impairment, and urinary incontinence, with enlarged ventricles of the brain but normal or slightly elevated cerebrospinal fluid (CSF) pressure [1], [2]. Currently there is no pathological hallmark for iNPH [3]. Studies suggesting some potential genetic background of iNPH have been published [4], [5]. The present treatment of choice in iNPH is CSF diversion with an implanted shunt that relieves or even reverses the symptoms. Various procedures to evaluate CSF dynamics in patients with possible iNPH are used to identify those who could benefit from CSF shunting. These include the CSF tap test, external lumbar drainage test, infusion tests, and intraventricular or intracranial pressure (ICP) monitoring [6]–[9]. The most frequent differential diagnoses of iNPH are atypical Alzheimer's disease (AD) and vascular dementia [8], .

AD is characterized by the hallmark lesions of amyloid-β (Aβ) plaques and neurofibrillary tangles composed of hyperphosphorylated tau (HPτ) in the brain of patients with amnestic cognitive decline [11]–[13]. The amyloid cascade hypothesis states that Aβ starts to accumulate decades before the clinical manifestations of AD [14], [15]. *In vivo*, Aβ can be detected directly with brain biopsy [8], [16], or indirectly by observing low levels of Aβ in CSF [17]. Fibrillar Aβ can also be evaluated by positron emission tomography (PET) utilizing e.g. the ^11^C-labeled Pittsburgh compound B [18] or [^18^F]flutemetamol [19].

Although common pathways for iNPH and AD have been proposed [20], the findings in genetic [21] and Aβ studies [8] suggest differences in etiologies of the two diseases. Aβ and HPτ in the CSF may help to differentiate iNPH and AD patient groups or detect comorbid AD in iNPH [17]. In addition, these biomarkers have shown a potency to predict response to shunt in iNPH [22], [23].

Aβ originates from a cell membrane-spanning protein, amyloid precursor protein (APP), which has diverse roles in normal neuronal function [24]. Soluble APP alpha (sAPPα) and beta (sAPPβ) result from the cleavage of APP by α- and β-secretases, respectively. Low CSF levels of sAPP isoforms have been reported in post-stroke patients and iNPH compared to AD and normal healthy controls [25]–[28]. In addition, sAPPα has shown a marked prognostic value for cognitive performance following shunt surgery [27], and subsequent increase of ventricular sAPP-levels has been noted in shunt-responders [28].

Abnormal levels of proinflammatory cytokines, such as interleukins (IL), interferon-gamma (IFN-γ), monocyte chemoattractant protein-1 (MCP-1), and tumor necrosis factor-alpha (TNF-α), in CSF have been noted in various diseases of the nervous system, including AD [29]. In iNPH, several proinflammatory cytokines have been studied, but none of them has proven to be useful in diagnostics [30]. Lower levels of IL-1β in NPH compared to AD was reported in a single paper [31], while increased levels of IL-4 and IL-10 were reported in patients with NPH compared to healthy individuals in another study, but no significant difference was seen between NPH and other dementias [32]. No differences were found between NPH and AD or healthy controls in studies comparing the levels of IL-8, IL-10, IL-12 (p40 and p70), IFN-γ, and transforming growth factor-β1 (TGF- β1) [28], [33]. However, iNPH patients did show increased levels of MCP-1 compared to healthy individuals [28]. Prior to treatment, higher TNF-α concentrations (and subsequent normalization after shunting) in CSF were observed in NPH patients compared to healthy controls in a single study [34]; however, these results did not replicate in a more recent study with solely idiopathic NPH patients [35].

Elevated levels of neurofilament light protein (NFL) in the CSF, indicating neuronal death and axonal loss, have been found in iNPH and secondary NPH in several studies [28], [36]–[39]. Increased levels of myelin basic protein (MBP) in the CSF is a well-established biomarker for demyelination and myelin damage in the central nervous system [40]. Furthermore, elevated levels of MBP have been reported in NPH [41].

To our knowledge, studies assessing the association between proinflammatory cytokines and biomarkers of neuronal damage in CSF, and cortical brain biopsy have not been published to date.

### Objectives

The objectives of the current study were:

1. to determine the levels of AD-related biomarkers (Aβ42, p-tau, total tau), non-AD-related Aβ isoforms (Aβ38, Aβ40), sAPP isoforms (sAPPα, sAPPβ), proinflammatory cytokines (IL 1β, 2, 4, 5, 8, 10, 12p70, and 13, IFN-γ, MCP-1, TNF-α) and biomarkers of neuronal damage (NFL, MBP) in lumbar and ventricular CSF, and how they correlate,

2. study the relationship between the CSF biomarkers and cortical brain biopsy,

3. assess the diagnostic and prognostic value of the CSF biomarkers in iNPH and AD.

## Methods

### Ethics statement

This study was approved by the Kuopio University Hospital (KUH) Research Ethical Committee, The Finnish National Supervisory Authority for Welfare and Health, and The Finnish Ministry of Social Affairs and Health. All participants or their proxies gave a written, informed consent prior to participation in the study. If the clinician suspected dementia to significantly affect the capacity of the patient to consent, a next of kin, caretakers or guardians consented on the behalf of participants. When a consent was obtained from a participant's proxy, the patients own opinion was inquired and considered, and no patients were recruited against their will.

### Kuopio NPH Registry and Protocol

Neurosurgery of KUH solely provides full-time acute and elective neurosurgical services for the KUH catchment population in Middle and Eastern Finland. In addition, the KUH area contains four central hospitals with neurological units and catchment areas of their own [21].

Patients fulfilling the following criteria were further assessed in KUH Neurosurgery as possible NPH patients: (1) primary evaluation and examination by a neurologist indicating NPH; (2) one to three symptoms suggestive of NPH (gait disorder, cognitive impairment, urinary incontinence); and (3) NPH related brain imaging findings (enlarged ventricles (Evans' index>0.3) together with obliterated cortical sulci). The diagnostic workup protocol of KUH Neurosurgery for possible NPH included a clinical examination, CT or MRI scan, and 24 h intraventricular ICP monitoring together with a frontal cortical brain biopsy. Kuopio NPH Registry (www.uef.fi/nph) consists of all evaluated possible NPH patients from the KUH catchment population since 1993 [21].

The ICP criteria for the shunt treatment in iNPH patients were (1) a basal ICP pressure between 10 and 20 mmHg continuously, or (2) the presence of A-waves or more than 30% B-waves during the 24 h monitoring, when basal pressure was between 5 and 10 mmHg [21].

### Study population

Altogether 102 patients, 51 men and 51 women, with a median age of 74.6 years (range 47–87 years) from the Kuopio NPH Registry with cortical brain biopsy, *APOE* genotype, and a ventricular CSF sample available were included in the study (Table 1). 63 patients were diagnosed with iNPH according to the protocol above, and were shunted with ventriculoperitoneral shunt (PS Medical medium pressure or adjustable valve). The clinical response to shunt was evaluated at 2–3 months after surgery, and any subjective or objective improvement in patient gait, memory or urinary continence was graded as a positive shunt response. Clinical AD was diagnosed according to a protocol described earlier [8], [10], [21] in 26 patients (including 10 patients with initial/primary diagnosis of iNPH) in a median follow-up time of 2.3 years (range 0.2–6.2 years).

**Table 1:** Characteristics, brain biopsy findings, and *APOE-ε4* statuses of 102 patients with possible NPH.

	Possible NPH (*n* = 102)				
	Final clinical diagnosis of iNPH (*n* = 53)			No diagnosis of iNPH (*n* = 39)	
Characteristics	Shunt responder	Shunt nonresponder	Mixed iNPH + AD	AD	Other
*n*	48	5	10	16	23
Age (years) (median (range))	72.7 (63.8–87.3)	82.8 (79.9–86.2)	78.4 (69.8–86.7)	78.3 (54.1–85.7)	70.6 (47.1–81.7)
Women (*n* (%))	23 (48)	3 (60)	3 (30)	10 (63)	23 (52)
Follow-up time (years) (median (range))	2.51 (0.82–6.22)	2.16 (1.27–3.21)	1.95 (0.99–3.60)	1.88 (0.35–3.06)	2.35 (0.19–4.88)
Leading symptom, *n* (%)					
Gait disorder	29 (60)	3 (60)	2 (20)	3 (19)	8 (35)
Memory impairment	9 (19)	0 (0)	5 (50)	10 (63)	5 (22)
Other	2 (4)	0 (0)	0 (0)	1 (6)	6 (26)
Undefined	8 (17)	2 (40)	3 (30)	2 (13)	4 (17)
Immunoreactivity (*n* (%))					
Aβ − HPτ −	24 (50)	2 (40)	3 (30)	4 (25)	15 (65)
Aβ + HPτ −	17 (35)	2 (40)	2 (20)	6 (38)	6 (26)
Aβ + HPτ +	4 (8)	1 (20)	5 (50)	6 (38)	1 (4)
Aβ − HPτ +	3 (6)	0 (0)	0 (0)	0 (0)	1 (4)
*APOE*-ε4 carriers (*n* (%))	9 (19)	2 (40)	7 (70)	7 (44)	4 (17)

Abreviations: *APOE*, apolipoprotein E gene; CSF, cerebrospinal fluid; NPH, normal pressure hydrocephalus; iNPH, idiopathic NPH; AD, Alzheimer's disease; Aβ, amyloid beta protein; HPτ, hyperphosphorylated tau protein.

### Immunohistochemistry and histological evaluation

Biopsy samples representing frontal cortex and subcortical white matter were stained with hematoxylin-eosin and immunostained with monoclonal antibodies directed to Aβ (6F/3D) and HPτ (AT8) as described earlier in detail [8], [21]. Positive Aβ immunostain was further quantified and reported as ratio of area covered by Aβ to total area of cortex in the biopsy as described earlier [17].

### *APOE* genotyping

DNA was extracted from venous blood using commercial kit according to manufacturer's protocol (Illustra Blood GenomicPrep Mini Spin Kit, GE Healthcare, Little Chalfont, UK). A standard PCR method was used in the *APOE* genotyping [21], [42].

### CSF samples and biomarker analyses

The ventricular CSF samples were collected immediately after the placement of intraventricular catheter (first 1 mL discarded) in the ICP measurement procedure. In addition, a lumbar CSF sample was available in 49 patients. The lumbar samples were obtained through a lumbar puncture prior to the ICP measurement protocol (12 patients) or 24–48 hours after introducing the ventricular catheter (37 patients).

Levels of AD biomarkers (Aβ42, p-tau, total tau) were measured from the CSF samples using commercial ELISA kits (Innotest β-amyloid_1–42_, Innotest Phosphotau_(181P)_, Innotest Tau-Ag, Innogenetics, Ghent, Belgium) according to the manufacturer's protocol at validated laboratory in Neurology (www.uef.fi/neuro), University of Eastern Finland, Kuopio, Finland as described earlier [17].

Aβ isoforms (Aβ38, Aβ40), sAPP isoforms (sAPPα, sAPPβ), and the proinflammatory cytokines (IL 1β, 2, 4, 5, 8, 10, 12p70, and 13, IFN-γ, MCP-1, TNF-α) were analyzed utilizing commercially available multiplexed assays (Meso Scale Discovery, Gaithersburg, MD, USA) [28], [43], and NFL and MBP concentrations were measured using commercial ELISA kits (NF-Light, UmanDiagnostics, Umeå, Sweden, and ACTIVE MBP, Diagnostic Systems Laboratories, Webster, TX, USA, respectively). All analyses were performed according to the manufacturers' protocols by board-certified laboratory technicians at the Clinical Neurochemistry Laboratory, Sahlgrenska University Hospital, Mölndal. Each set of biomarker measurements was performed on one day, using one batch of reagents.

All clinical, immunohistochemical, and laboratory analyses were performed blinded to the result information of each other.

### Statistical analyses

Nonparametric Kruskal–Wallis H and Mann–Whitney U tests were used for comparing CSF levels of measured biomarkers between different groups, and the Wilcoxon signed-rank test for comparisons of two related samples. To define the correlation between different proinflammatory cytokines, Pearson correlation with Bonferroni correction (*k* = 55) was applied.

Patients with tauopathy but no amyloid (*n* = 4) were excluded from the analyses of different biomarkers in relation to biopsy findings, and iNPH patients with co-morbid AD (*n* = 10) and iNPH patients with negative shunt-response (*n* = 5) from the analyses comparing true iNPH patients to non-iNPH patients. Some cytokines were below the lower limit of detection of the assays and graded as zero concentration in statistical analyses. One patient had an insufficient CSF sample for the analysis of sAPPs, and another for the analysis of Aβ isoforms Aβ38 and Aβ40.

IBM SPSS Statistics for Mac (version 19.0.0.2, IBM, Armonk, NY, USA) was used in the statistical analyses. The level of significance was set at *p*<0.05.

## Results

### Aβ and sAPP isoforms

**Figure 1:**
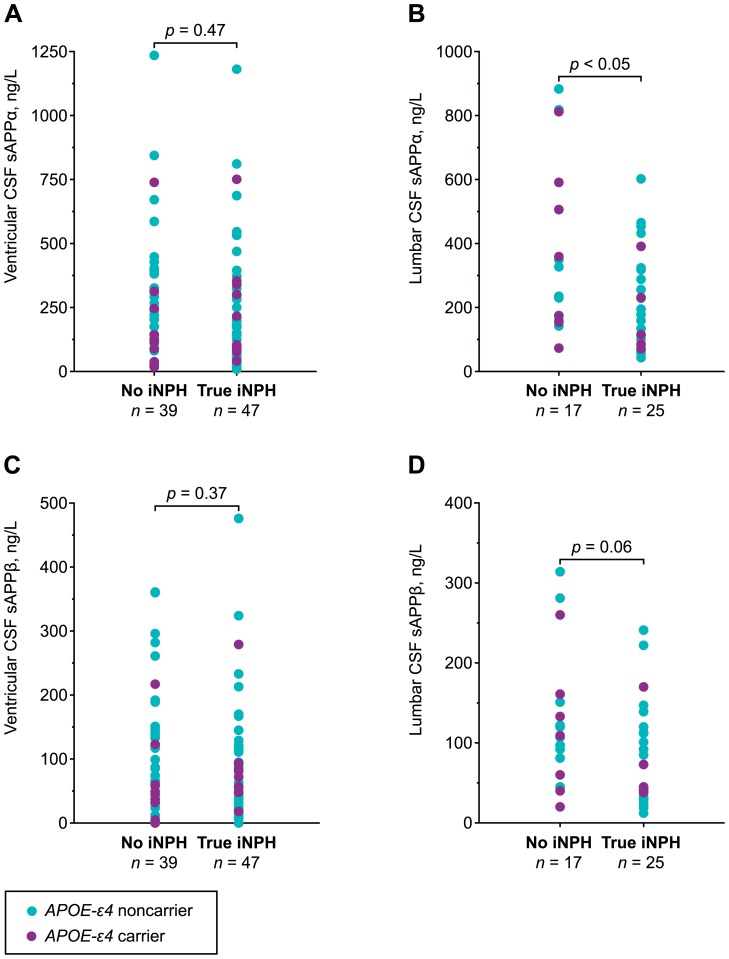
CSF sAPP isoforms in true iNPH patients and patients with no diagnosis of iNPH. Scatterplots of soluble amyloid precursor protein alpha (sAPPα) and beta (sAPPβ) concentrations in ventricular (A and C) and lumbar (B and D) cerebrospinal fluid (CSF) in patients with no diagnosis of idiopathic normal pressure hydrocephalus (iNPH) and patients with true (shunt-responsive) iNPH are presented. Cases are color-labeled according to their *APOE-ε4* status. *P*-values were determined using a Mann–Whitney U test.

Lumbar CSF levels of sAPPα were significantly lower (*p*<0.05) in patients with iNPH and a positive shunt reponse compared to non-iNPH patients, while sAPPβ showed a similar tendency (*p* = 0.06, Table 2, Figure 1B and 1D). However, no such association was seen for sAPP isoforms in ventricular CSF samples (*p* = 0.37–0.47, Table 2, Figure 1A and 1C). In iNPH patients, a tendency towards lower levels of sAPPα and sAPPβ were observed (*p* = 0.23–0.49) in shunt-responders compared to nonresponders in ventricular and lumbar CSF (Table 2).

**Table 2:** CSF biomarker levels of 102 patients with possible NPH.

	Possible NPH (*n* = 102)				
	Final clinical diagnosis of iNPH (*n* = 53)			No diagnosis of iNPH (*n* = 39)	
CSF Biomarker*	Shunt responder	Shunt nonresponder	Mixed iNPH + AD	AD	Other
*n*	48	5	10	16	23
Aβ42					
Lumbar	587 (141)	563 (241)	487 (256)	584 (248)	667 (181)
Ventricular	476 (203)	428 (250)	450 (134)	422 (259)	542 (271)
P-tau 181					
Lumbar	35.3 (15.5)	38.0 (14.8)	41.1 (22.1)	47.1 (13.6)	43.2 (15.5)
Ventricular	77.1 (51.7)	50.4 (14.3)	93.1 (39.9)	81.3 (51.2)	91.8 (133)
Total tau					
Lumbar	239 (156)	255 (121)	211 (135)	294 (164)	252 (98.9)
Ventricular	1,210 (1186)	562 (443)	1,500 (1,320)	1,361 (1687)	1,698 (3,313)
sAPPα					
Lumbar	217 (156)	472 (560)	350 (137)	325 (238)	424 (300)
Ventricular	237 (241)	374 (484)	261 (193)	223 (216)	304 (284)
sAPPβ					
Lumbar	84.3 (63.4)	193 (200)	102 (44.9)	108 (67.9)	158 (101)
Ventricular	88.3 (92.3)	169 (235)	110 (92.6)	83.6 (81.7)	123 (109)
IL-8					
Lumbar	1,101 (2,440)	318 (374)	85.7 (73.1)	461 (592)	466 (420)
Ventricular	20.2 (20.8)	33.0 (51.3)	23.0 (5.76)	85.9 (286)	19.1 (11.0)
IL-8 ratio					
Lumbar/ventricular	132 (446)	55.0 (84.1)	3.05 (2.01)	51.7 (105)	20.9 (14.5)
MCP-1					
Lumbar	3,398 (2,865)	1,876 (766)	785 (515)	2,618 (2,881)	3,262 (2,061)
Ventricular	748 (280)	1,096 (688)	784 (187)	819 (436)	758 (264)
TNF-α					
Lumbar	4.38 (7.80)	1.88 (1.40)	0.50 (0.86)	2.56 (3.45)	2.76 (2.58)
Ventricular	0.31 (0.62)	0.28 (0.63)	0.25 (0.55)	0.57 (1.16)	0.00 (0.00)
NFL					
Lumbar	2,511 (1,798)	6,545 (6,242)	2,153 (830)	2,007 (867)	1,399 (538)
Ventricular	886 (681)	6,692 (9,723)	1,993 (1,715)	1,567 (2,152)	1,010 (864)
MBP					
Lumbar	117 (170)	27.0 (9.93)	17.5 (–)	49.9 (57.7)	90.6 (61.7)
Ventricular	8.22 (12.1)	12.9 (19.2)	50.5 (73.5)	9.03 (18.8)	21.2 (30.8)

Abreviations: CSF, cerebrospinal fluid; Aβ42, amyloid beta 1–42; p-tau 181, tau phosphorylated at threonine 181; sAPP, soluable amyloid precursor protein; IL-8, interleukin 8; MCP-1, monocyte chemoattractant protein-1; TNF-α, tumor necrosis factor-alpha; NFL, neurofilament light protein; MBP, myelin basic protein.

*Mean (SD) CSF concentrations in ng/L.

**Figure 2:**
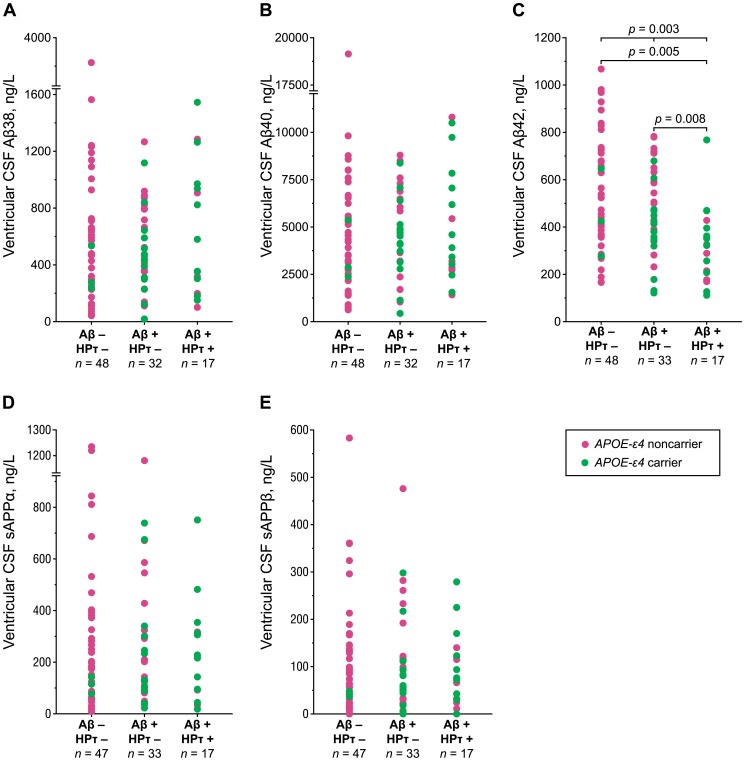
CSF Aβ and sAPP isoforms in different brain biopsy groups. Scatterplots of amyloid beta 1–38 (Aβ38) (A), Aβ40 (B), Aβ42 (C), soluble amyloid precursor protein alpha (sAPPα) (D), and beta (sAPPβ) (E) in groups of positive/negative Aβ and hyperphosphorylated tau (HPτ) immunoreactivity in brain biopsy are presented. Cases are color-labeled according to their *APOE-ε4* status. *P*-values were determined using a Kruskal–Wallis H test and post-hoc Mann–Whitney U test with Bonferroni correction. Only statistically significant *p*-values are shown.

**Figure 3:**
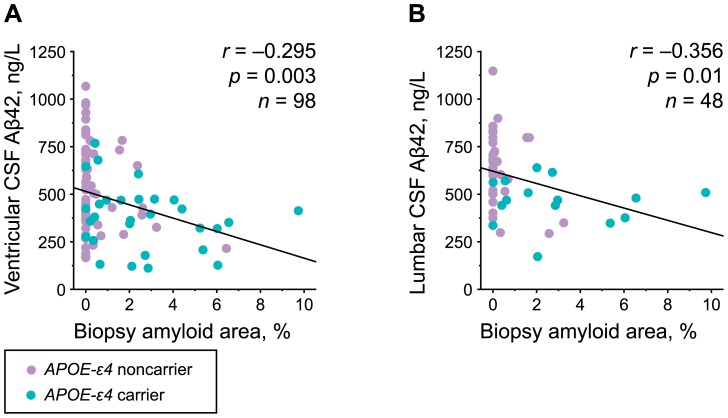
CSF Aβ42 in relation to amyloid-β deposits in cortical brain biopsies. Scatterplots of ventricular (A) and lumbar (B) cerebrospinal fluid (CSF) amyloid beta 1–42 (Aβ42) levels in relation to the percentage of Aβ area in frontal cortical brain biopsies are presented. Cases are color-labeled according to their *APOE-ε4* status. Correlation coefficients and *P*-values were determined using Pearson correlation.

Ventricular CSF levels of Aβ42 differed (*p* = 0.003) between different brain biopsy groups (Table 3, Figure 2C). Patients with positive Aβ and HPτ immunoreactivity in the cortical brain biopsy showed significantly lower CSF levels of Aβ42 compared to the Aβ positive and HPτ negative group (post hoc *p* = 0.008), and to the Aβ and HPτ negative group (post hoc *p* = 0.005, Table 3, Figure 2C). Similar associations were seen in lumbar samples (data not shown). Quantified Aβ load showed a negative correlation with the levels of CSF Aβ42 in ventricular (Pearson's *r* = −0.295, *p* = 0.003) and lumbar (Pearson's *r* = −0.356, *p* = 0.01) samples (Figure 3). However, the CSF levels of other Aβ isoforms (Aβ38, Aβ40) and sAPP isoforms (sAPPα, sAPPβ) did not correlate (*p* = 0.59–0.95) with the presence of Aβ or HPτ in the biopsy (Table 3, Figure 2A–B and 2D–E). Furthermore, there was no correlation between the levels of sAPP isoforms in the CSF and Aβ load in cortical brain biopsy (*p* = 0.84–0.92). There were no statistically significant differences between the levels of CSF Aβ or tau biomarkers in shunt-responding and nonresponding iNPH patients.

**Table 3:** Lumbar and ventricular CSF biomarkers in different brain biopsy findings.

	Immunoreactivity				
Variable	Aβ− HPτ−	Aβ+ HPτ−	Aβ− HPτ+	Aβ+ HPτ+	Total
*n*					
Lumbar	23	17	1	8	49
Ventricular	48	33	4	17	102
Aβ38*					
Lumbar	682 (370)	754 (365)	923 (–)	849 (352)	739 (360)
Ventricular	583 (612)	533 (305)	564 (544)	615 (464)	572 (499)
Aβ40*					
Lumbar	6,105 (3,007)	6,170 (2,157)	8143 (–)	6,735 (1,916)	6,272 (2,521)
Ventricular	4,544 (3,162)	4,768 (2,203)	4,858 (3,598)	5,101 (3,082)	4,721 (2,855)
Aβ42*					
Lumbar	641 (184)	596 (167)	758 (–)	393 (108)	588 (187)
Ventricular	529 (240)	485 (188)	463 (327)	320 (162)	477 (225)
P-tau 181*					
Lumbar	37.8 (15.9)	37.0 (15.0)	45.6 (–)	48.9 (16.0)	39.5 (15.7)
Ventricular	81.3 (97.3)	73.6 (47.3)	83.5 (23.3)	95.8 (57.1)	81.3 (75.5)
Total tau*					
Lumbar	238 (139)	246 (164)	287 (–)	296 (127)	251 (143)
Ventricular	1,423 (2,386)	1,054 (1,104)	1,387 (415)	1,652 (1,970)	1,340 (1,924)
sAPPα*					
Lumbar	300 (287)	288 (211)	178 (–)	323 (261)	298 (251)
Ventricular	266 (283)	264 (263)	179 (190)	249 (184)	259 (256)
sAPPβ*					
Lumbar	112 (106)	110 (77.0)	92 (–)	106 (75.8)	110 (89.5)
Ventricular	104 (116)	105 (107)	69.5 (65.2)	95.3 (76.6)	102 (105)
IL-8*					
Lumbar	852 (2,299)	435 (732)	197 (–)	1,218 (1,901)	754 (1,792)
Ventricular	43.5 (165)	22.8 (24.3)	16.9 (8.04)	15.6 (10.0)	31.1 (114)
IL-8 ratio**					
Lumbar/ventricular	154 (525)	36.9 (51.3)	9.12 (–)	154 (133)	112 (370)
MCP-1*					
Lumbar	2,994 (2,628)	2,519 (2,354)	3,059 (–)	3,637 (3,323)	2,935 (2,602)
Ventricular	767 (391)	820 (304)	704 (191)	771 (165)	782 (327)
TNF-α*					
Lumbar	2.77 (4.94)	2.55 (2.85)	1.27 (–)	6.88 (11.2)	3.34 (5.91)
Ventricular	0.28 (0.74)	0.22 (0.59)	0.36 (0.72)	0.34 (0.67)	0.27 (0.67)
NFL*					
Lumbar	2,216 (2,044)	3,135 (3,073)	1,180 (–)	2,479 (1,987)	2,557 (2,419)
Ventricular	1,127 (1,385)	2,082 (4,153)	618 (448)	1,213 (1,307)	1,430 (2,615)
MBP*					
Lumbar	71.5 (77.6)	82.2 (71.6)	48.9 (–)	160 (275)	89.9 (131)
Ventricular	12.7 (24.2)	17.1 (33.5)	30.5 (23.5)	9.65 (18.7)	14.4 (26.8)

Abreviations: CSF, cerebrospinal fluid; Aβ, amyloid beta protein; HPτ, hyperphosphorylated τ protein; p-tau 181, tau phosphorylated at threonine 181; sAPP, soluable amyloid precursor protein; IL-8, interleukin 8; MCP-1, monocyte chemoattractant protein-1; TNF-α, tumor necrosis factor-alpha; NFL, neurofilament light protein; MBP, myelin basic protein.

*Mean (SD) CSF concentrations in ng/L.

**Mean (SD). Only cases with CSF sample obtained after 24 h ICP monitoring included (*n* = 37).

### Proinflammatory cytokines

Several cytokines were present at concentrations below the lower limit of detection of the assay (Table S1). All tested proinflammatory cytokines showed positive correlations between each other in ventricular (Table S1) and lumbar (Table S2) CSF samples. All ventricular and lumbar CSF IL-8 samples and all but one ventricular CSF MCP-1 sample were above the lower limit of detection, and the two cytokines and TNF-α were chosen for further analyses from the proinflammatory cytokines measured.

**Figure 4:**
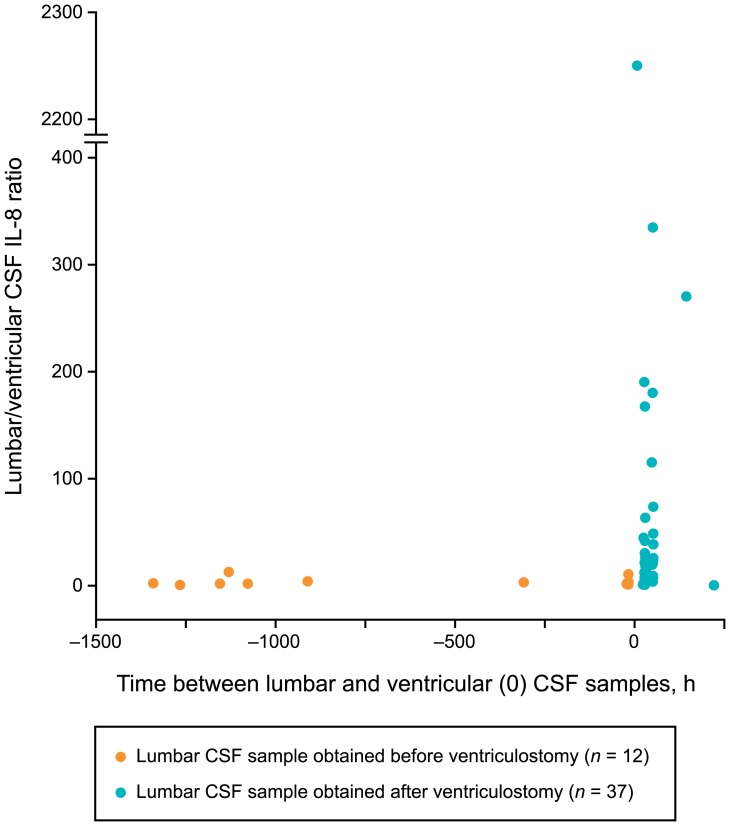
Lumbar/ventricular CSF IL-8 ratio in relation to time between the CSF samples. Scatterplot of lumbar/ventricular cerebrospinal fluid (CSF) interleukin 8 (IL-8) ratio in individual cases in relation to the time difference between lumbar and ventricular samples is presented. Cases are color-labeled according to whether lumbar CSF sample was collected before or after the ventricular sample.

Lumbar CSF samples showed higher IL-8 levels compared to ventricular samples (*p*<0.001). Moreover, lumbar/ventricular CSF IL-8 ratios (*p*<0.001) were significantly higher in samples collected after the ventriculostomy and ICP measurement compared to those collected before the procedure (Figure 4).

The levels of tested proinflammatory cytokines in the CSF showed no association to the presence of Aβ or HPτ in brain biopsy, diagnosis of iNPH or AD, or shunt response in iNPH patients.

### Biomarkers of neuronal damage

There was no significant relation of CSF NFL or MBP levels to the brain biopsy findings or to the diagnosis of iNPH or AD. In iNPH, a tendency (*p* = 0.05) towards lower ventricular CSF NFL values was seen in shunt-responders (Table 2).

## Discussion

This is the first study to explore Aβ and sAPP isoforms, proinflammatory cytokines, and biomarkers of neuronal damage in the CSF in conjunction with cortical brain biopsy. The major finding in the current study was the demonstration of the independent role of sAPPα in iNPH, which is not explained by cortical Aβ pathology.

In iNPH, decreased levels of sAPP isoforms in lumbar CSF have been reported in previous studies [26]–[28]. As predicted, the level of lumbar CSF sAPPα was lower, whereas sAPPβ showed a similar trend in patients with shunt-responsive iNPH compared to non-iNPH patients in our patient cohort. Interestingly, in ventricular samples no association with iNPH was noted, which could be explained by the ventriculostomy procedure as an invasive sample collection method or by concentration of proteins in lumbar CSF. However, the reason for unaffected ventricular levels of sAPP isoforms are obscure as the ventricular CSF could be expected to reflect the periventricular metabolism better than lumbar CSF. The pathobiological role of sAPP isoforms in iNPH seems to be unconnected to the amyloidogenic pathway as there was no correlation between sAPP isoform levels in the CSF and Aβ load in the cortical brain biopsy. The reason for the lowering of sAPP isoform levels in untreated iNPH remains unclear. However, as the levels are restored upon successful shunt treatment it has been hypothesized that the lowering may reflect metabolic impairment in brain tissue affected by iNPH [28]. In any case, our data suggest that the observed sAPP isoform level changes are independent of Aβ pathology or are so early in the cascade that they do not reflect current tissue pathology.

As expected, CSF levels of Aβ42 showed a negative association and correlation to Aβ load in the brain, as published earlier [17], and as suggested by amyloid-imaging studies [44]. In contrast, no such association was seen between the CSF levels of Aβ38 or Aβ40 with positive Aβ or HPτ immunoreactivity in the cortical brain biopsy. Our findings support the non-amyloidogenic role of Aβ38 and Aβ40 in the living human brain.

As predicted, AD patients had lower Aβ42 and higher p-tau levels in the CSF compared to non-AD patients, although the differences did not reach statistical significance. One explanation to this is that non-AD patients show similar CSF Aβ42 and p-tau findings as biomarkers of comorbid AD tissue pathology without clinical dementia of Alzheimer's type. As no pathological hallmark lesions have been identified in iNPH [3], the role of Aβ and tau in iNPH remain elusive. However, there are patients with mixed pathologies i.e., patients with AD-related pathology and later dementia but still initial objective response for shunt surgery [10]. Interestingly, patients with iNPH + AD had lowest levels of CSF Aβ42, and highest frequency of *APOE*-ε4 carriers. Eighty percent (8/10) of these patients showed a favourable response to shunt treatment.

In contrast to two previous studies [22], [23], we found no prognostic potential in the levels of CSF Aβ42 or tau in shunted iNPH patients. The differences in the results may be explained by the different sample collection time (most the samples in the current study were collected after ventriculostomy). It should also be noted that cited studies included fewer patients.

In iNPH, reports of abnormal levels of proinflammatory cytokines (IL-1β, IL-4, IL-10, MCP-1, TNF-α) in the CSF has been published [28], [31], [32], [34], while contradictory findings in studies comparing proinflammatory cytokines (IL-8, IL-10, IL-12 (p40 and p70), IFN-γ, TNF-α, TGF- β1) have also been reported [28], [33], [35]. In the current study,we made an attempt to measure a wide panel of different proinflammatory cytokines from ventricular and lumbar CSF, and positive correlations between cytokines were seen. However, we also noted that most of the cytokines are present in CSF at concentrations that are close to or below the lower limit of detection of the assay. In fact, these low concentrations, which are technically challenging to measure, may explain some of the varying results in the published literature. Here, we focused on the cytokines that could be robustly quantified in at least a subset of samples, i.e., IL-8. No association of proinflammatory cytokines in the CSF with the diagnosis of iNPH or AD or the presence of Aβ or HPτ in brain biopsy was seen. In patients with iNPH, proinflammatory cytokines did not show prognostic value in shunt surgery. Consequently, our data suggest the role of neuroinflammation in iNPH and AD to be of little importance. Instead, an inflammatory response was seen in lumbar CSF samples collected after the ventriculostomy and ICP measurement. In addition, increased CSF tau and p-tau levels were observed in lumbar samples obtained after ventriculostomy as reported in earlier studies [17], [39]. In consequence, levels of biomarkers in post-ventriculostomy lumbar CSF may not reflect the true values of the biomarkers in these patients.

Previous studies have reported increased levels of CSF NFL in NPH [28], [36]–[39]. In our cohort, NFL showed higher lumbar CSF levels in patients with shunt-responsive iNPH compared to non-iNPH patients (Table 2), but not to a significant degree. Interestingly, iNPH patients with positive shunt response showed a tendency towards lower NFL levels in ventricular CSF compared to shunt-nonresponsive iNPH patients. As NFL reflects subcortical axonal damage, perhaps high NFL could represent more severe and less recovering injury in the hydrocephalic brain.

The strengths of the current study include: a large NPH cohort evaluated by cortical brain biopsy, utilization of ventricular and lumbar CSF samples in the analyses, a wide panel of tested CSF biomarkers, and evaluation of clinical outcome and other dementing disorders in the follow-up. The limitations of this study included: a limited number of patients with no shunt response, systematic assessment of shunt response only at 2–3 months, dichotomised shunt response scale, lack of validated objective outcome measures, and lumbar CSF sample available only in half of the cases. It is obvious that at least in prospective research setting shunted patients should be followed-up for a significantly longer time, and validated outcome measures should be utilized. Five patients with diagnostic findings suggesting iNPH did not respond to shunt – possibly due to comorbidities or misdiagnosis, and thus these patients were excluded from the comparisons of (‘true’) iNPH patients and non-iNPH patients in addition to the iNPH patients who were diagnosed with comorbid AD in the follow-up.

More basic science and clinical studies evaluating the biology and potential role as diagnostic and prognostic biomarker of sAPPα and β are needed in the future.

To conclude, the role of sAPP isoforms in iNPH seems to be unconnected to the Aβ cascade pathway, but rather may be explained by a metabolic failure or ischemia in the brain. No elevations in the levels of proinflammatory cytokines in the CSF were observed in the different diagnostic groups. Consequently, neuroinflammation in iNPH and AD require further study. None of the tested CSF biomarkes showed a potency to discriminate between iNPH and non-iNPH patients or shunt-responders and nonresponders in iNPH in clinical setting.

## Supporting Information

Table S1 **Correlations of proinflammatory cytokines in lumbar CSF.**
(PDF)

Table S2 **Correlations of proinflammatory cytokines in ventricular CSF.**
(PDF)
