# Supplementary material for: Cerebrospinal Fluid Biomarker and Brain Biopsy Findings in Idiopathic Normal Pressure Hydrocephalus
Source: PLoS One. 2014 Mar 17;9(3):e91974. doi: 10.1371/journal.pone.0091974 (PMC3956805; doi:10.1371/journal.pone.0091974)
Supplement: Table S1 — Correlations of proinflammatory cytokines in lumbar CSF. (PDF) [file pone.0091974.s001.pdf]

Table S1. Correlations of proinflammatory cytokines in lumbar CSF.

		IL-1β	IL-2	IL-4	IL-5	IL-8	IL-10	IL-12p70	IL-13	MCP-1	IFN-γ	TNF-α
	**Pearson's r**	1										
**IL-1β**	***P***											
	**No.**	49										
	**Pearson's r**	0.827	1									
**IL-2**	***P***	<0.001										
	**No.**	49	49									
	**Pearson's r**	0.088	0.085	1								
**IL-4**	***P***	>0.99	>0.99									
	**No.**	49	49	49								
	**Pearson's r**	0.783	0.839	0.087	1							
**IL-5**	***P***	<0.001	<0.001	>0.99								
	**No.**	49	49	49	49							
	**Pearson's r**	0.687	0.731	0.344	0.775	1						
**IL-8**	***P***	<0.001	<0.001	0.86	<0.001							
	**No.**	49	49	49	49	49						
	**Pearson's r**	0.784	0.881	0.193	0.872	0.931	1					
**IL-10**	***P***	<0.001	<0.001	>0.99	<0.001	<0.001						
	**No.**	49	49	49	49	49	49					
	**Pearson's r**	0.387	0.398	0.266	0.519	0.720	0.611	1				
**IL-12p70**	***P***	0.33	0.26	>0.99	<0.01	<0.001	<0.001					
	**No.**	49	49	49	49	49	49	49				
	**Pearson's r**	0.727	0.741	0.245	0.836	0.919	0.900	0.713	1			
**IL-13**	***P***	<0.001	<0.001	>0.99	<0.001	<0.001	<0.001	<0.001				
	**No.**	49	49	49	49	49	49	49	49			
	**Pearson's r**	0.520	0.572	0.031	0.787	0.677	0.769	0.422	0.722	1		
**MCP-1**	***P***	<0.01	<0.001	>0.99	<0.001	<0.001	<0.001	0.14	<0.001			
	**No.**	49	49	49	49	49	49	49	49	49		
	**Pearson's r**	0.688	0.700	0.340	0.716	0.909	0.841	0.749	0.827	0.556	1	
**IFN-γ**	***P***	<0.001	<0.001	0.92	<0.001	<0.001	<0.001	<0.001	<0.001	<0.01		
	**No.**	49	49	49	49	49	49	49	49	49	49	
	**Pearson's r**	0.806	0.917	0.149	0.903	0.856	0.925	0.568	0.886	0.687	0.820	1
**TNF-α**	***P***	<0.001	<0.001	>0.99	<0.001	<0.001	<0.001	<0.01	<0.001	<0.001	<0.001	
	**No.**	49	49	49	49	49	49	49	49	49	49	49

Abreviations: CSF, cerebrospinal fluid; IL, interleukin; MCP-1, monocyte chemoattractant protein-1; IFN-γ, interferon-gamma; TNF-α, tumor necrosis factor-alpha.

All *P* values are Bonferroni corrected (k = 55).
