# Supplementary material for: Cerebrospinal Fluid Biomarker and Brain Biopsy Findings in Idiopathic Normal Pressure Hydrocephalus
Source: PLoS One. 2014 Mar 17;9(3):e91974. doi: 10.1371/journal.pone.0091974 (PMC3956805; doi:10.1371/journal.pone.0091974)
Supplement: Table S2 — Correlations of proinflammatory cytokines in ventricular CSF. (PDF) [file pone.0091974.s002.pdf]

Table S2. Correlations of proinflammatory cytokines in ventricular CSF.

		IL-1β	IL-2	IL-4	IL-5	IL-8	IL-10	IL-12p70	IL-13	MCP-1	IFN-γ	TNF-α
	**Pearson's r**	1										
**IL-1β**	***P***											
	**No.**	102										
	**Pearson's r**	0.492	1									
**IL-2**	***P***	<0.001										
	**No.**	102	102									
	**Pearson's r**	0.178	0.165	1								
**IL-4**	***P***	>0.99	>0.99									
	**No.**	102	102	102								
	**Pearson's r**	0.512	0.826	–0.010	1							
**IL-5**	***P***	<0.001	<0.001	>0.99								
	**No.**	102	102	102	102							
	**Pearson's r**	0.495	0.863	–0.010	0.986	1						
**IL-8**	***P***	<0.001	<0.001	>0.99	<0.001							
	**No.**	102	102	102	102	102						
	**Pearson's r**	0.518	0.927	0.076	0.868	0.914	1					
**IL-10**	***P***	<0.001	<0.001	>0.99	<0.001	<0.001						
	**No.**	102	102	102	102	102	102					
	**Pearson's r**	0.496	0.677	0.556	0.498	0.497	0.562	1				
**IL-12p70**	***P***	<0.001	<0.001	<0.001	<0.001	<0.001	<0.001					
	**No.**	102	102	102	102	102	102	102				
	**Pearson's r**	0.522	0.737	0.434	0.630	0.632	0.662	0.847	1			
**IL-13**	***P***	<0.001	<0.001	<0.001	<0.001	<0.001	<0.001	<0.001				
	**No.**	102	102	102	102	102	102	102	102			
	**Pearson's r**	0.228	0.490	–0.055	0.425	0.493	0.570	0.213	0.240	1		
**MCP-1**	***P***	>0.99	<0.001	>0.99	<0.001	<0.001	<0.001	>0.99	0.86			
	**No.**	101	101	101	101	101	101	101	101	101		
	**Pearson's r**	0.278	0.362	0.305	0.157	0.153	0.304	0.495	0.399	0.128	1	
**IFN-γ**	***P***	0.26	0.01	0.10	>0.99	>0.99	0.10	<0.001	<0.01	>0.99		
	**No.**	102	102	102	102	102	102	102	102	101	102	
	**Pearson's r**	0.639	0.658	0.109	0.601	0.629	0.738	0.460	0.501	0.392	0.559	1
**TNF-α**	***P***	<0.001	<0.001	>0.99	<0.001	<0.001	<0.001	<0.001	<0.001	<0.01	<0.001	
	**No.**	102	102	102	102	102	102	102	102	101	102	102

Abreviations: CSF, cerebrospinal fluid; IL, interleukin; MCP-1, monocyte chemoattractant protein-1; IFN-γ, interferon-gamma; TNF-α, tumor necrosis factor-alpha.

All *P* values are Bonferroni corrected (k = 55).
